# Catheter Intervention in a Patient with Intracranial Aneurysms and Glanzmann Thrombasthenia Caused by a Novel Homozygous Likely Pathogenic Variant in the *ITGA2B* Gene

**DOI:** 10.3390/diseases12070136

**Published:** 2024-06-27

**Authors:** Doris Boeckelmann, Lara von Dobeneck, Hans Henkes, Hermann Eichler, Hannah Glonnegger, Barbara Zieger

**Affiliations:** 1 Department of Pediatrics and Adolescent Medicine, Division of Pediatric Hematology and Oncology, Medical Center—University of Freiburg, 79106 Freiburg, Germany; 2 Neuroradiological Clinic, Klinikum Stuttgart, 70174 Stuttgart, Germany; 3 Institute for Diagnostic and Interventional Radiology and Neuroradiology, University Hospital Essen, 45147 Essen, Germany; 4 Institute of Clinical Hemostaseology and Transfusion Medicine, Saarland University and University Hospital, 66424 Homburg, Germany

**Keywords:** inherited platelet disorder, Glanzmann Thrombasthenia (GT), *ITGA2B* gene, catheter intervention

## Abstract

Glanzmann Thrombasthenia (GT) is an inherited platelet disorder caused by defects in platelet integrin α_IIb_β_3_ (GPIIb/IIIa), which is a platelet receptor essential for the binding of fibrinogen. This can lead to severe bleeding, especially after trauma or perioperatively, and to microcytic anemia because of chronic blood loss. We report on a 40-year-old female patient with extensive bleeding complications and platelet antibody formation who presented in Homburg and Freiburg for extensive platelet function analyses and molecular genetic analyses. According to platelet aggregometry, the patient had previously been diagnosed with Glanzmann Thrombasthenia (GT). In addition, an MRI scan had been performed due to an unsteady gait and had revealed bilateral para-ophthalmic aneurysms of both internal carotid arteries (ICAs). Assuming a 5% rupture risk per 5 years for each aneurysm, the patient was offered and accepted endovascular treatment. Next-generation sequencing (NGS) panel analysis identified a previously undescribed homozygous one-base-pair deletion in *ITGA2B*, which leads to a loss of function of the α_IIb_-subunit of the receptor. This case illustrates the difficulties that can arise regarding the treatment of patients with rare platelet bleeding disorders, and supports the importance of continuous medical care by a specialized hemophilia center for these patients.

## 1. Introduction

Glanzmann Thrombasthenia (GT) is a rare autosomal recessively inherited platelet function defect caused by quantitative or qualitative abnormalities of the platelet integrin α_IIb_β_3_ complex. The two genes encoding α_IIb_β_3_, *ITGA2B* and *ITGB3,* are both located on chromosome 17q21; *ITGA2B* is much larger consisting of 30 exons in contrary to *ITGB3* with 15 exons. Pathogenic variants in both genes have been described to be associated with GT [1,2]. Meanwhile, about 290 mutations in *ITGA2B* and 229 mutations in *ITGB3* have been listed in the Human Genetic Mutation Database (https://www.hgmd.cf.ac.uk, accessed on 1 November 2023). Due to impaired binding of its main ligand, fibrinogen, platelets fail to aggregate, resulting in inadequate primary hemostasis [3]. Symptoms range from moderate to severe mucocutaneous bleeding, such as purpura, epistaxis, and gum bleeding [4]. In affected females, menorrhagia is common and pregnancy-related bleeding is a feared complication [5]. The prevalence is estimated to be one in one million, with a higher prevalence in populations where consanguinity is more common [5,6].

Here, we present the case of a 40-year-old woman with Glanzmann Thrombasthenia caused by a novel likely pathogenic variant in the α_IIb_-subunit encoding gene *ITGA2B* and platelet alloantibody formation, who also suffered from intracranial aneurysms.

## 2. Case Presentation

A 40-year-old woman was referred to our outpatient clinics for platelet flow cytometry and genetic analyses. She presented with multiple hematomas on her trunk and extremities. Her parents are cousins who originated from western Turkey. Although she had been diagnosed with Glanzmann Thrombasthenia at a very young age, she was not yet treated in a specialized hemophilia center, and neither flow cytometry nor genetic analysis had been performed.

The patient’s medical history revealed a history of severe bleeding. She suffered from menorrhagia, which presumably led to her microcytic anemia, and had received platelet concentrates multiple times as a young adult as a precaution before dental surgery. During her pregnancy in 2010, alloantibodies to glycoprotein α_IIb_β_3_ were detected in the patient’s blood. In the 38th week of pregnancy, cardiotocography revealed fetal bradycardia and an emergency caesarean section was performed in another hospital. Because the patient developed severe bleeding problems, she received two red cell units, two pooled platelet concentrates, tranexamic acid, and desmopressin intraoperatively. Two drainages were placed. Three platelet concentrates were transfused in the following days, resulting in a first-time transfusion reaction. Curettage was performed. However, the vaginal bleeding did not stop. Therefore, endometrial coagulation, intracavitary balloon catheter placement, and finally operative hysteroscopy were performed. The patient’s condition slowly improved.

### 2.1. Platelet Function Analyses and Molecular Genetic Analysis

**Figure 1:**
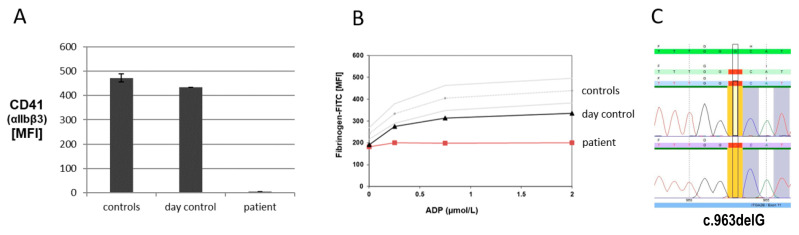
Platelet flow cytometry and Sanger sequencing for the patient with Glanzmann Thrombasthenia. (**A**) Expression of CD41 (α_IIb_β_3_) is absent on the patient’s platelets. (**B**) Fibrinogen binding after activation with different concentrations of ADP is absent compared to the day control and healthy controls (mean and standard deviation of 20 controls), indicating Glanzmann Thrombasthenia. (**C**) In exon 11 of the *ITGA2B* gene a homozygous one-base-pair deletion at c.963 was detected by NGS and confirmed by direct sequencing.

In March 2021, she was found to have cerebral aneurysms and the question of bleeding management during neuroradiologic procedures arose. She was referred to the University Hospitals in Homburg and Freiburg for comprehensive platelet characterization. Her blood analysis revealed hypochromic anemia and a platelet count of 198 × 10^9^/L (normal range 150–400 × 10^9^/L). Platelet aggregometry showed no aggregation after stimulation with collagen (2 and 10 µg/mL), ADP (4 and 10 µmol/L), and epinephrine (8 µmol/L) (norm > 70%), and reduced (42%, Norm > 70%) agglutination after stimulation with ristocetin (1.2 mg/mL). Flow cytometric assessment of platelets was performed using FACSCalibur (Becton Dickinson, Heidelberg, Germany). Diluted PRP aliquots (5 × 10^7^/mL) were fixed and stained with a FITC-labeled monoclonal surface antibody against glycoproteins (GPs) CD41 (GPIIb/IIIa-complex, integrin αIIbβ3), CD42a (GPIX), and CD42b (GPIb) (Coulter, Immunotech, Marseille, France). FITC-labeled anti-VWF (Bio-Rad AbD Serorech, Puchheim, Germany) and Alexa Fluor 488-labeled anti-fibrinogen (Invitrogen, Waltham, MA, USA) were used to stain the platelets. In the presence of 1.25 mM Gly-Pro-Arg-Pro (Bachem, Bubendorf, Switzerland), diluted PRP (5 × 10^7^ platelets/mL) was stimulated with different concentrations of thrombin (0, 0.05, 0.1, 0.2, 0.5, and 1U/mL; Siemens Healthineers, Marburg, Germany) to conduct the CD62 and CD63 expression analyses. Additionally, the platelets were stained with monoclonal FITC-labeled anti-CD62 (P-selectin) and anti-CD63 antibodies (lysosomal-associated membrane glycoprotein 3, LAMP-3; Immunotech, Marseille, France). Data of patients and controls (day control and 20 independent measurements from 10 controls as mean ± standard error of the mean, SEM) were analyzed. Flow cytometry showed the absence of expression of CD41 (integrin α_IIb_β_3_) and severely impaired fibrinogen binding after ADP activation (Figure 1A,B). Furthermore, a MAIPA (monoclonal antibody immobilization of platelet antigens) assay was performed, which identified platelet-specific alloantibodies to integrin α_IIb_β_3_ (moderately positive) and antibodies to HLA class I antigens (weakly positive).

A molecular genetic analysis was performed using a custom gene panel for inherited platelet disorders (Nextera Rapid Custom Enrichment, Illumina, San Diego, CA, USA) established in our department, followed by sequencing on a MiSeq (Illumina). This revealed a homozygous one-base-pair deletion (NM_000419:c.963delG) in exon 11 of the *ITGA2B* gene encoding the integrin subunit α_IIb._ The deletion results in a frameshift and a premature stop codon (p.His322Ilefs*57). The variant is annotated in dbSNP (v156, rs2048619871) but not in the population database gnomAD (v2.1.1) nor in mutation/variant databases, such as HGMD or ClinVar (Access April 2024). The finding was validated by Sanger sequencing (Figure 1C). The alteration is located in the β-propeller-region of integrin α_IIb_, which is a domain important for fibrinogen binding [7]. While integrin α_IIb_ normally consists of 1039 amino acids, this truncation results in a remarkably smaller protein of 378 amino acids with a frameshift and termination prior to the transmembrane domain. No other variant was found in either *ITGA2B* or *ITGB3*. Sequence Pilot (JSI medical systems) copy number variation analysis did not show any deviation either. According to ACMG Standards and Guidelines for the interpretation of sequence variants, as well as the newly adapted version solely for *ITGA2B* and *ITGB3* from 2021, this variant is classified as “likely pathogenic” as the combined evidence meets the required criteria (ACMG: PVS1, PM2) [7,8].

### 2.2. Catheter Intervention

The patient had originally presented to our specialized facility in Stuttgart for consultation and possible endovascular treatment of her intracranial aneurysms. The patient was given an estimated risk of rupture of 5% per 5 years per aneurysm with resulting morbidity and mortality of >35%. The risk of major complications from endovascular treatment was estimated to be 3% per aneurysm. We expected potential bleeding risks to be related more to arterial access to the groin artery than to the actual treatment of the two aneurysms. In the first session, the paraophthalmic aneurysm of the right internal carotid artery (ICA) was catheterized and occluded with detachable coils. For treatment of the wide-neck aneurysm of the left ICA, we anticipated the need to fixate the detachable coils in the aneurysm with a stent or, even better, with a flow diverter. Such vascular implants with an antithrombogenic coating have been available since 2019. In patients with normal platelet function, uncoated implants require dual antiplatelet therapy (e.g., aspirin and ticagrelor). Stents and flow diverter devices with a hydrophilic coating (HPC) can be implanted under the influence of only one antiplatelet drug (e.g., prasugrel). In this patient, antiplatelet therapy was omitted due to Glanzmann Thrombasthenia. At the same time, it seemed at least possible that the detected platelet dysfunction was so pronounced that the implantation of a flow diverter with HPC would not lead to thrombus formation on the foreign surface even without medication.

Immediately after coil occlusion of the right aneurysm, a flow diverter (p48MW HPC 2 mm/12 mm; WallabyPhenox) was implanted in the main stem of the right external carotid artery (ECA). This was performed under the assumptions that (1) a possible embolic occlusion of the ECA would be tolerated without damage and that (2) the possible absence of thrombotic occlusion of the flow diverter in the right ECA would lead to the expectation that a corresponding flow diverter in the left ICA would also be tolerated without medication. The second treatment was performed eleven weeks after the first. Injection of the right ICA and ECA confirmed sufficient coil occlusion of the aneurysm and patency of the flow diverter. The left ICA aneurysm was partially filled with seven coils and covered with a flow diverter (p64MW HPC 3.5 mm/18 mm, WallabyPhenox). Repeated MRI/MRA follow-ups confirmed the elimination of both aneurysms and the patency of both flow diverters. There were no complications related to femoral access or anterior cerebral circulation. The patient’s clinical status was mRS 0 before treatment and has remained unchanged since. In the 2.5 years since the initial procedure, no thrombotic occlusion has occurred in either of the two implanted flow diverters with HPC.

## 3. Discussion

In the patient discussed in this study, alloantibodies against integrin α_IIb_β_3_ were first detected during pregnancy. According to Botero et al., in up to 70% of pregnant women who suffer from GT, integrin α_IIb_β_3_ or HLA alloantibodies have been identified [5]. It remains unclear whether this patient developed them as a result of multiple prophylactic platelet concentrates because of her dental treatments or because of her pregnancy. Fiore et al. noted that premature truncation mutations carry a high risk for platelet antibody formation. They also made the assumption that transfusion therapy early in life leads to a higher chance of antibody formation [9]. This means that our patient had a high risk of developing platelet alloantibodies.

Therefore, if possible, not only the severity of bleeding symptoms but also the type of mutation and the patient’s age should be taken into account when considering the transfusion of platelet concentrates. The presence of platelet antibodies carries a risk of refractoriness to platelet transfusions in critical situations, leading to severe perioperative bleeding. When HLA class I antibodies are present, as in this patient, only compatible concentrates should be administered, which may not always be available. According to Botero et al., platelet transfusions in GT patients should be reserved for severe bleeding and major surgery [5]. Treatment with antifibrinolytics and/or rFVIIa may reduce the need for platelet transfusions. Accordingly, the prophylactic use of platelet concentrates should be further discussed in the future due to the increased risk of antibody development. This should be considered especially in young women planning to have a child, as platelet alloantibodies can cross the placental barrier and may cause fetal thrombocytopenia [10]. Since 2019, rFVIIa has been approved for the treatment of GT even without the detection of anti-integrin α_IIb_β_3_ alloantibodies when platelet concentrates are unavailable and/or in the presence of HLA antibodies with previous or current platelet refractoriness (EMA/CHMP/690988/2018). It can be a good alternative to transfusions if the patient responds adequately to aFVII. In this context, it should be noted that rFVIIa did not work very well during the cesarean section in this patient, according to her medical history. Another treatment option in patients with GT and very severe/life-threatening bleeding symptoms may be hematopoietic stem cell transplantation (HSCT) [11]; however, this therapy can have other complications and should, therefore, be intensively evaluated.

## 4. Conclusions

Medical care in a specialized haemophilia center is very important for patients with inherited platelet disorders to ensure comprehensive diagnosis and optimal treatment, as well as intensive patient education. The identification of novel genetic variants helps to gain novel knowledge regarding platelet physiology and pathophysiology. GT patients with intracerebral aneurysm need special bleeding management if catheter intervention is needed.

## Data Availability

Due to privacy, data are available upon request, subject to evaluation by the corresponding author.
